# Frankfurters Manufactured with Valorized Grape Pomace as a Substitute of Nitrifying Salts

**DOI:** 10.3390/foods14030391

**Published:** 2025-01-24

**Authors:** María Jesús Martín-Mateos, Jonathan Delgado-Adámez, María Díaz-Ponce, David Tejerina, María Rosario Ramírez-Bernabé

**Affiliations:** Centro de Investigaciones Científicas y Tecnológicas de Extremadura (CICYTEX), Instituto Tecnológico Agroalimentario de Extremadura (INTAEX), Avda Adolfo Suárez s/n, 06071 Badajoz, Spain; mariajesus.martinmat@juntaex.es (M.J.M.-M.); david.tejerin@juntaex.es (D.T.)

**Keywords:** grape pomace, polyphenols, antioxidants, frankfurters, nitrites

## Abstract

This study investigated the use of grape/wine pomace as a potential substitute for nitrifying salts in the production and preservation of frankfurters. Red wine pomace (RWP) from *Tempranillo* and white wine pomace (WWP) from *Cayetana* grapes were added to frankfurters made with Iberian pig backfat—an underutilized fat rich in oleic acid—at two levels (0.5% and 3% *w*/*w*). These new formulations were compared with a control (containing only meat, salt, and spices) and a commercial formulation containing nitrites and ascorbic acid. Analyses were conducted immediately after production and following 45 days of refrigerated storage to evaluate microbiological, color, physicochemical, and textural changes in the frankfurters. The addition of pomace slightly reduced the pH of the frankfurters but did not affect microbial counts during the manufacturing process. Frankfurters with pomace displayed a similar color to the control but showed lower redness compared to the commercial formulation with nitrites. Importantly, pomace reduced lipid and protein oxidation during production and storage. The reduction in lipid oxidation due to the pomace was comparable to the effect of nitrites and ascorbic acid. Furthermore, pomace effectively reduced protein oxidation, unlike nitrites and ascorbic acid, which primarily targeted lipid oxidation. Significant differences in texture were observed between commercial frankfurters and those containing pomace. Despite these variations in the appearance and the texture, the strong protective effect of pomace against oxidative reactions highlights its potential as a natural alternative to synthetic additives, offering a promising solution for the meat industry.

## 1. Introduction

The winemaking industry is one of the largest global producers of by-products, presenting a significant environmental challenge. Grape pomace is the most abundant by-product of winemaking, accounting for 62% of the total by-products generated [1,2]. It consists of a mixture of seeds, stems, skins, stalks, and pulp residues. The significance of grape pomace lies in its richness in polyphenols [3], which exhibit antimicrobial and antioxidant properties [4]. Furthermore, the bioactive compounds in grape pomace have numerous health benefits, including reducing the risk of certain degenerative diseases [5]. The composition of grape pomace varies depending on factors such as grape variety, ripeness, climatic conditions, and winemaking practices [6,7]. Additionally, red and white grape pomaces differ significantly due to variations in the chemical composition of the grapes and the winemaking processes. In red wine production, the grape skins remain in contact with the juice during fermentation, facilitating the transfer of phenolic compounds that contribute to color and other attributes. Conversely, in traditional white wine production, only the grape juice (must) undergoes fermentation, as the skins are removed beforehand. As a result, white grape pomace contains higher sugar and moisture levels but contains no alcohol [1].

Traditionally, wine pomace has been used for the production of distilled alcohol [8]. Other common uses include its application as fertilizer or animal feed [9]. However, recent studies have explored alternative applications for wine pomace.

Several investigations have focused on extracting bioactive compounds from pomace, which can be utilized in the pharmaceutical, cosmetic, and food industries [10]. In the food industry, research has emphasized the development of value-added products [1], such as bioactive compound extracts (primarily phenols), the recovery of tartaric acid, and the production of flours. These components can serve as antioxidants, fortifying agents, colorants, and antimicrobial additives in food products. Such extracts have been applied in various food matrices, including meat and fish products.

For effectively valorized by-products like grape pomace, it is essential to stabilize the product microbiologically and enzymatically before its incorporation into food matrices [11]. This ensures that the valorized ingredient is safe, has a low microbial load, and possesses an extended shelf life. Stabilization is particularly important for stationary by-products like wine pomace. Furthermore, the valorized by-product must exhibit low enzymatic activity, such as polyphenol oxidase, to preserve phenolic compounds in the ingredient [12,13]. High hydrostatic pressure (HHP) is a non-thermal processing technology used to inactivate microorganisms while minimizing chemical reactions in foods [14]. Although HHP preserves phenolic compounds, it does not inhibit enzymatic activity, including polyphenol oxidase, during storage. This enzymatic activity leads to a reduction in polyphenol contents over time, thereby shortening the shelf life of the valorized by-product [12,13]. However, applying thermal blanching prior to HHP effectively inhibits polyphenol oxidase and stabilizes the polyphenol contents during storage [11]. The primary advantage of using HHP for by-product valorization is its ability to preserve phenolic compounds in wine pomace [12,13,15]. Additionally, this technology enables the integral utilization of all pomace components—seeds, stems, skins, stalks, and pulp—without the need for solvents or the generation of waste. This makes HHP a green technology, aligning with sustainable practices for by-product valorization.

Frankfurter-type sausages are widely consumed meat products, offering significant opportunities for innovation. Their production typically involves the addition of nitrites, which play a key role in color development. The fixation of the characteristic pink color is one of the most noticeable effects of nitrite addition and is considered a critical attribute for consumer acceptance [16]. In addition to color formation, nitrites provide antimicrobial and antioxidant benefits by protecting lipids from rancidity and contributing to the flavor and aroma of cured products [17]. However, the use of nitrites and nitrates is controversial due to their toxicological effects, as they are linked to the formation of N-nitrosamines, compounds with carcinogenic, teratogenic, and mutagenic properties [11]. In recent years, researchers have focused on reducing or substituting nitrites in the meat products industry [18]. One promising approach involves the use of plant-based ingredients with antimicrobial and antioxidant properties as replacements for nitrites [19]. For example, Armenteros et al. [20] evaluated the effect of adding phenolic extracts to frankfurters manufactured without traditional additives like sodium ascorbate and nitrites. Their study concluded that combining phenolic extracts with traditional additives effectively enhanced the oxidative stability of the frankfurters without altering their color or texture properties.

The use of by-products rich in phenolic compounds, such as grape pomace, has been investigated as a substitute for nitrates in dry-cured sausages [21]. The inclusion of this by-product effectively inhibited microbial growth and extended shelf life. Similarly, the addition of grape seed flour to frankfurter formulations reduced oxidation, thanks to its potent antioxidant properties [22]. Additionally, grape seed extracts have been shown to limit lipid oxidation in frankfurters [23]. A recent study by Martín Mateos et al. [11] evaluated the inclusion of valorized white grape pomace treated with HHP in pork burgers. The pomace ingredient reduced lipid and protein oxidation development during storage, although it presented limited antimicrobial and color-protective effects. To date, the use of fresh valorized red and white grape pomace by-products has not been evaluated in frankfurters.

On the other hand, the Iberian pig production system is predominantly focused on dry-cured products, such as hams and shoulders. However, this industry generates significant quantities of by-products, like subcutaneous fat, whose primary use—the production of dry-cured fermented sausages—does not fully utilize the amounts produced. Notably, fat from pigs raised under the Montanera system features a healthy fatty acid profile, with high levels of linoleic acid [3], making it a valuable ingredient for the production of other meat products. Therefore, the main objective of this study was to evaluate the application of an ingredient obtained from an integral valorization process of grape pomace as a substitute for nitrifying salts, in order to improve the preservation of pork frankfurters.

## 2. Materials and Methods

### 2.1. Material

#### 2.1.1. Raw Materials for the Production of Grape Pomace

Red wine pomace (RWP) from the *Tempranillo* variety was collected from the Santa Marta de los Barros Cooperative (Badajoz, Spain). White wine pomace (WWP) from *Cayetana* grapes was provided by a local wine manufacturer in Alburquerque (Badajoz, Spain). Approximately 10 kg of each pomace was collected, vacuum-packaged in 1–2 kg plastic bags (OptiDure™ ODA7005, oxygen permeability: 10 cm^3^ m^−2^, 24 h^−1^ at 0% relative humidity; Cryovac, Madrid, Spain), and vacuum-sealed at −0.8 bar using a Henkovac Proeco vacuum sealer (Henkovac International, Hertogenbosch, The Netherlands). The packages were stored at −80 °C until further processing.

#### 2.1.2. Manufacturing Process of Grape Pomace

Previous studies [12,13] examined the effects of high hydrostatic pressure (HHP) on red and white wine pomace and revealed that HHP did not reduce polyphenol oxidase enzyme (PPO) activity. To inactivate PPO, thermal blanching was performed before the HHP treatment. The thermal blanching process involved steam treatment at 103 °C, using an Exhauster unit equipped with upper and lower stainless steel mesh belts with variable speed drag (1 min residence time). These parameters were based on preliminary experiments, which demonstrated complete PPO inactivation after 1 min of thermal treatment, while preserving phenolic compounds content [11]. After blanching, the pomace was frozen at −18 °C. Once fully frozen, it was ground using a Thermomix (Vorwerk, Germany) at maximum speed for 1–2 min. This grind–freeze cycle was repeated three times until a fine powder was obtained. The powdered pomace was vacuum-packaged and processed using HHP at 600 MPa for 5 min in semi-industrial equipment (Hiperbaric 6000/55; Burgos, Spain) with a 55 L capacity and an initial water temperature of 16 °C. The HHP conditions were chosen based on prior studies, which showed extended stability (at least nine months under refrigeration) of the processed pomace, enabling its use in the meat industry year-round despite its seasonal availability. The valorized grape pomace (processed through blanching, milling, and HHP) was vacuum-packaged and stored at −80 °C until use. It was incorporated into pork frankfurters at various concentrations.

#### 2.1.3. Manufacturing, Packaging, and Storage of Frankfurters

**Figure 1:**
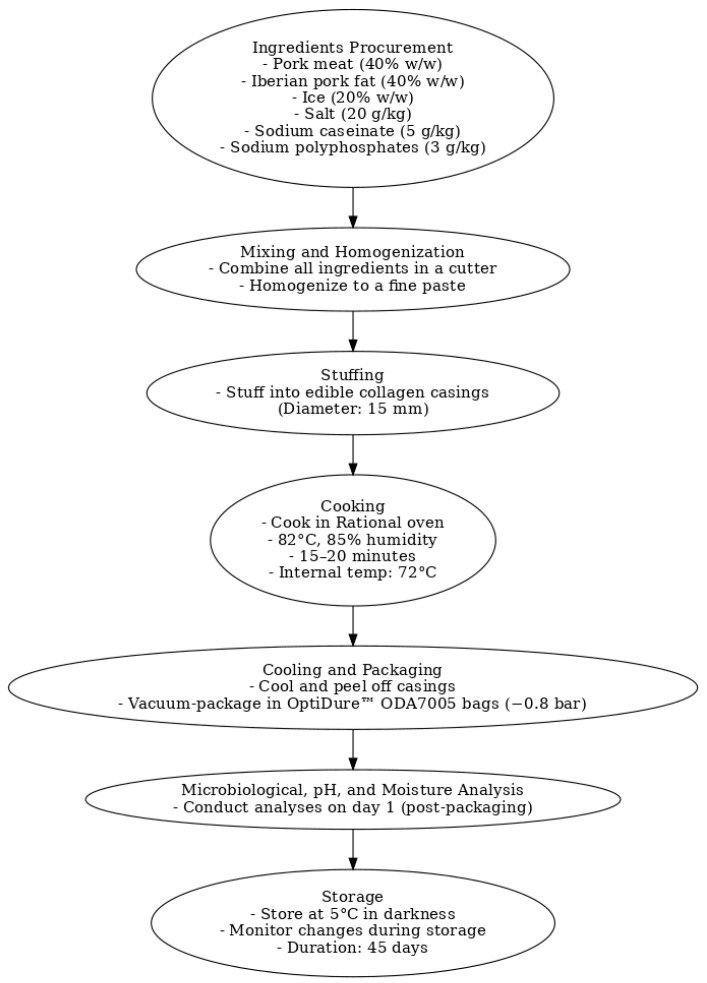
Flow chart of the manufacturing, packaging, and storage processes for frankfurter sausages.

Figure 1 represents the flow chart of the manufacturing, packaging, and storage processes for frankfurter sausages. A standard recipe was followed to prepare the frankfurters, consisting of pork meat (40% *w*/*w*), Iberian pork fat (40% *w*/*w*), and ice (20% *w*/*w*). These ingredients were locally sourced. The pork was obtained from commercial pig breeds, while the Iberian pork backfat came from 100% Iberian pigs raised outdoors using the traditional *Montanera* rearing system. Each batch included additional ingredients: salt (20 g kg^−1^), sodium caseinate (5 g kg^−1^), and sodium polyphosphates (3 g kg^−1^). The mixture was homogenized in a cutter (Mainca, CM-21, Granollers, Barcelona, Spain) until a fine paste was achieved, then stuffed into edible collagen casings (15 mm diameter). The sausages were cooked in a Rational oven (Combimaster Plus, Cornellà de LLobregat, Barcelona, Spain) at 82 °C and 85% humidity for 15–20 min until their internal temperature reached 72 °C. After cooling, the sausages were peeled and vacuum-packaged in OptiDure™ ODA7005 bags (−0.8 bar, Cryovac, Madrid, Spain). Microbiological, pH, and moisture analyses were performed the day after packaging (day 1). The sausages were stored at 5 °C in darkness to monitor changes during storage.

### 2.2. Experimental Design

**Figure 2:**
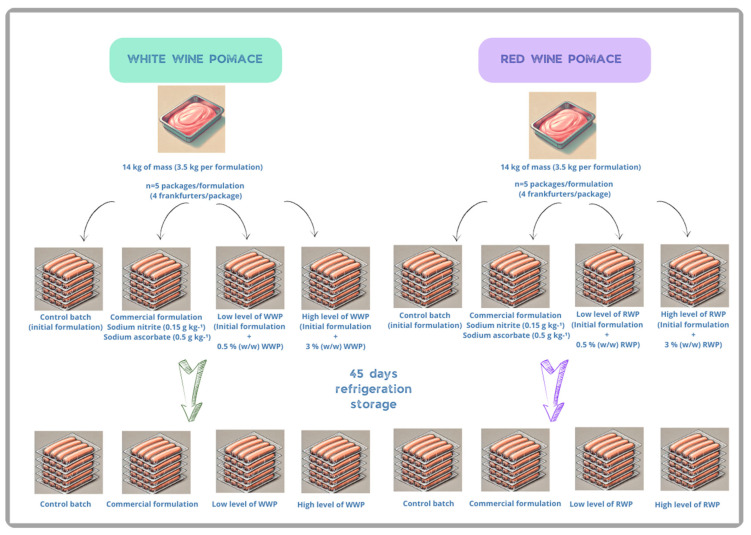
Experimental plan of the manufacturing, packaging, and storage processes for frankfurter sausages.

Figure 2 shows the experimental design for the production and storage of frankfurters. The initial formulation was used as the control batch. A commercial formulation was also prepared, containing sodium nitrite (0.15 g kg^−1^) and sodium ascorbate (0.5 g kg^−1^). To evaluate the effect of the pomace ingredient, two levels (0.5% and 3% *w*/*w*) of RWP and WWP were added to the control formulations. In the frankfurters, 0.5% (*w*/*w*) was considered a low level of pomace, while 3% (*w*/*w*) was considered a high level. These levels were selected based on sensory analyses conducted in previous studies.

The experiment to evaluate the RWP was independent to the WWP, so two experiments were performed. For each experiment, a 14 kg mass (3.5 kg per formulation (3.5 × 4)) was prepared. Ten packages per formulation were evaluated (five for day 1 and five for day 45). Each package included four sausages (measuring 15–18 cm in length) with the same formulation. Therefore, for the evaluation of frankfurters made using RWP, a total of 20 packages were analyzed on day 1 (after manufacturing, T0), and the other 20 packages were analyzed after 45 days of refrigerated storage (T1). One analysis per package was performed (taking a homogenous sample of 2–3 sausages). A similar scheme was followed for the evaluation of frankfurters made using WWP.

### 2.3. Methods

#### 2.3.1. Physico-Chemical Characterization of Grape Pomace and Frankfurters

The composition of the initial pomace was analyzed in three independent vacuum-packaged bags. For the evaluation of the frankfurters, five packages of sausages per formulation were analyzed. Moisture and protein analyses were determined according to Association of Analytical Communities (AOAC) Correcmethodology, the fat contents were assessed using the Folch method [24], and fiber contents were assessed using the modified Southgate method [25]; all of these were in wet base (WB). Likewise, the pH (Hanna instruments, Eibar, Guipuzcoa, Spain) and water activity (Novasina Labmaster, Novasina AG, Lachen, Switzerland) of the initial pomace were determined. Fatty acid methyl esters (FAMEs) from frankfurters were analyzed using an Agilent 6890 gas chromatograph (Agilent Technologies, Santa Clara, CA, USA), equipped with a flame ionization detector (FID). The results were expressed as a percentage of total fatty acid methyl esters. The phenolic compounds content was determined according to the methods by D’Arrigo et al. [13] and Ramírez et al. [12]. The capacity for radical scavenging of the samples was assessed using the ABTS (2,2′-azinobis(3-ethylbenzothiazoline-6-sulfonic acid) method. The initial absorbance value of 730 nm was compared with the absorbance obtained after 20 min of reaction. The results were expressed as mmol Trolox mL^−1^ on a calibration curve using different Trolox concentrations.

#### 2.3.2. Microbiological Analysis of Frankfurters

A 10 g sample was aseptically taken and homogenized with 90 mL of sterile peptone water using a laboratory blender (Stomacher R 400 Circulator, Seward Ltd., Worthing, UK) for 1 min. Serial decimal dilutions were then prepared in sterile peptone water, and 1 mL of each dilution was spread onto suitable culture media.

Microbial counts were determined as follows: Mesophilic aerobic bacteria were enumerated on plate count agar (Merck, 1.07881, Darmstadt, Germany) after incubation at 30 °C for 72 h. Psychrophilic bacteria were evaluated on plate count agar (Merck, 1.07881) after incubation at 7 °C for 10 days. Molds and yeasts were analyzed on a CG agar base (Merck, Darmstadt, Germany) supplemented with CGA selective supplement (Merck, Darmstadt, Germany), and incubated at 25 °C for 4–5 days. *E. coli* and total coliforms were detected on Chromocult agar (Merck, 1.10426) after incubation at 37 °C for 24–48 h. *Clostridium* spp. was cultured on tryptose sulfite cycloserine agar (Merck, 1.10235) and incubated at 37 °C for 24 h. *Staphylococcus aureus* were evaluated on Baird-Parker agar (Merck, 1.05406) after incubation at 37 °C for 24–48 h. *Salmonella* spp. and *L. monocytogenes* were analyzed following ISO 6579:1993 and ISO 11290-1:1996, respectively.

The results were expressed as Log_10_ CFU (colony-forming units) per gram. The detection limit for most techniques was 10 CFU g^−1^, except for *Clostridium* spp. and *S. aureus*, which had a detection limit of 100 CFU g^−1^. Additionally, the absence of *Salmonella* spp. and *L. monocytogenes* was confirmed in 25 g of sausage samples.

### 2.4. Instrumental Color

The color coordinates of lightness (L*), redness (a*; red/green axis), and yellowness (b*; yellow/blue axis) were analyzed in the CIE Lab color space. In addition, the hue angle was calculated (h° = tan^−1^ (b*/a*)), as well as the saturation index or chroma (C*) (C = (a* ^2^ + b* ^2^)^0.5^). In frankfurters, slices measuring one centimeter thick were prepared. Two readings were recorded (one on each side of the slice).

### 2.5. Oxidative Stability

Lipid oxidation was evaluated using the thiobarbituric acid reactive substances (TBA-RS) method, following Sørensen and Jørgensen’s protocol [26]. TBA-RS values were calculated using a standard curve prepared with 1,1,1,3-tetraethoxypropane (TEP), and results were expressed as milligrams of malondialdehyde per kilogram of sample (mg MDA kg^−1^).

Protein oxidation was measured by determining the carbonyl groups formed during the reaction with 2,4-dinitrophenylhydrazine (DNPH) in 2 N HCl, following the method of Oliver et al. [27]. Protein concentration was quantified spectrophotometrically at 280 nm, using bovine serum albumin (BSA) as the standard. Protein oxidation results were expressed as nanomols of carbonyls per milligram of protein (nmol carbonyls/mg protein).

The phenolic compound contents of the sausages was measured using the Folin–Ciocalteu reagent-based colorimetric assay. Firstly, 5 g of the sample were homogenized with the extraction solution (mili-Q water 80%; methanol 19.9%; citric acid 0.1%; *v*/*v*/*w*) and then the mixture was shaken and centrifuged. Subsequently, the methanol was evaporated, and the filtrate was then mixed with the Folin–Ciocalteu reagent (Sigma). After the addition of a saturated sodium carbonate solution (30%), the absorbance was measured at 760 nm using a Thermo-Evolution 201 spectrophotometer (USA). A calibration curve, using gallic acid as the reference standard, was generated. Total phenolic content was expressed as gallic acid equivalents per sample (wet base) (mg GAE 100 g^−1^).

### 2.6. Texture Profile Analysis

Textural characteristics of the frankfurters were determined using a TA XT-2i texture analyzer (Stable Micro Systems Ltd., Surrey, UK) and a texture profile analysis at 50% deformation (TPA50). The samples were prepared for TPA50 by creating five cubes (1 × 1 × 1 cm) from three frankfurters in each batch. Each portion was compressed to 50% of its initial height, using a 20 mm diameter flat plunger (P/20), and the following parameters were obtained: hardness (N cm^2^), springiness (cm), cohesiveness (dimensionless), gumminess (N × cm × s^2^), chewiness (N × cm × s^2^), and resilience (dimensionless).

### 2.7. Statistical Analysis

The results are expressed as means and standard deviations. For the pomace characterization, three independent samples were studied per batch (*n* = 3); however, for the frankfurters, five independent samples were studied per batch (*n* = 5). Student’s t-test was used to evaluate the differences between using white grape pomace and red grape pomace during the manufacturing of pork sausages (Table 1). Two one-way analyses of variance (ANOVAs) were performed on the data to discover the general effect of the formulation and storage. When the ANOVA showed a significant effect (*p* ≤ 0.05) due to the formulation, a Tukey test was performed.

## 3. Results and Discussion

The pH values of RWP and WWP were not significantly different (Table 1). Both pomaces displayed acidic pH levels, a characteristic typical of grape by-products, as observed in the previous literature [28]. The water activity (Aw) values also showed no significant differences between red and white wine pomace. Depending on the origin and the intensity of the pressure applied during the pressing operation, wine pomace can have significant differences in its moisture content [12,13]. The pH and Aw values were within the range observed in grape pomace from similar local grape varieties [12,13].

Moisture and protein content were similar in RWP and WWP; however, fiber and fat contents were significantly higher in RWP (Table 1). The composition of grape pomace varies significantly depending on factors such as the type of residue, grape variety, planting environment, and processing method. In red wine production, the entire mass of grapes is fermented, whereas, in white wine production, only the juice is fermented, resulting in a significant variation in its composition [29]. The protein content of wine pomace may range between 6% and 15% (dry matter) depending on grape variety and harvesting conditions [1], which is in line with our results (RWP: 10.1, WWP: 7.5 g 100 g^−1^ dry matter). The primary source of lipids in wine pomace comes from the seeds, and the lipid fraction presents an interesting fatty acids profile: it is rich in polyunsaturated fatty acids (PUFAs) and monounsaturated fatty acids (MUFAs) and has low levels of saturated fatty acids (SFAs) [1].

The phenolic compound content was higher in WWP than in RWP, and a similar trend was observed for antioxidant activity (Table 1). Previous studies have reported significant variability in the phenolic compound contents (PCC) of grape pomace, even within the same grape variety. For instance, fresh red grape pomace from the *Tempranillo* variety was found to contain 633 mg GAE 100 g^−1^ [13], which is higher than the values observed in the present study. In contrast, white wine pomace from varieties such as *Cayetana*, *Pardina*, and *Montúa* showed lower values (457 mg GAE 100 g^−1^ [12] than those reported here. Additionally, the valorization process applied to pomace could lead to modifications in PCC [11,30]. Polyphenolic compounds, including plant pigments such as anthocyanins and carotenoids, along with various minerals (e.g., copper, manganese, selenium, and zinc), vitamins (e.g., C, E, and A), and fiber compounds in grape pomace contribute to its antioxidant potential. Fiber compounds can form chemical bonds with phenolic substances, creating antioxidant dietary fibers that enhance radical scavenging capacity [29,31]. Antioxidants act by inhibiting or preventing oxidative reactions through mechanisms such as donating hydrogen atoms to lipid free radicals, neutralizing peroxide free radicals, and forming stable antioxidant-derived radicals [31]. In this respect, numerous studies have explored food products enriched with grape pomace, demonstrating their enhanced functional properties compared to conventional products [31].

The proximate composition of frankfurters containing RWP or WWP was not significantly altered by the inclusion of additives or pomace (Table 2). The frankfurters with RWP exhibited similar ranges of moisture, protein, and fat content, while those manufactured with WWP showed changes in fat content. In addition, the pH was reduced in the frankfurters with high levels of RWP and WWP and increased in the commercial ones. The acidic pH observed in frankfurters with high levels of pomace could be attributed to the low pH of the pomace, which is below four (Table 1). Similarly, Özvural and Vural [22] reported a decrease in the pH of frankfurters with increasing levels of grape seed flour in their formulation. Likewise, Abdelhakam et al. [32] observed a reduction in pH values in beef burgers as the addition of red grape pomace powder increased.

The fatty acid profile of the frankfurters (Table 2) reflects the characteristic composition of Iberian pork fat, which was used in their production. This is consistent with the high oleic acid content (51.0 ± 0.1) typical of this type of fat (see Supplementary Material, Table S1). The unique composition of Iberian pork fat results from the pigs’ diet, which primarily consists of acorns and grass [33]. The incorporation of Iberian pork backfat allows for the manufacturing of frankfurters with a high MUFA content, which can improve the fatty acids profile, thereby bringing it closer to fulfilling the recommendation to reduce SFAs and increase MUFAs [34] in order to prevent the development of cardiovascular diseases [35].

The fatty acids profile of frankfurters made with RWP was not affected by the formulation and only two fatty acids were modified, namely C18:1 and C18:3. Oleic acid levels were highest in the commercial group and lowest in frankfurters containing low levels of RWP. In WWP frankfurters, the control group exhibited higher SFA contents (C12:0, C14:0, C16:0, C17:0, and C18:0), while the commercial sausages and those formulated with pomace showed higher MUFA contents (C16:1, C18:1, and C20:1).

These changes in the profile align with findings in the literature regarding the fatty acid composition of grape seeds [1,36]. However, they do not align with expectations, given that RWP had a higher fat content than WWP.

Counts of mesophiles, psychrophiles, molds and yeasts, *S. aureus*, *Cl. perfringens*, total coliforms, and *E. coli* (Table 3) in frankfurters made with RWP and WWP were within an acceptable range for consumption, since counts were low or below the detection limit of the method. The frankfurters also presented with an absence of *Salmonella* spp. and *L. monocytogenes* within 25 g of product. The product was thermally treated at 70 °C for 20 min, and it demonstrated high stability. The addition of synthetic additives or grape pomace did not affect the initial counts of the control group, so they did not result in any advantages. However, the effect of additives or pomace during the storage process was not evaluated; it is likely that, at the moment, any effects they have would increase the shelf life and the safety of the product. Previous studies demonstrated the antimicrobial activity of grape (*Vitis vinifera* L) seed extracts against *E. coli* and *Listeria* [4]. The addition of the white grape pomace also resulted in an antimicrobial effect, as the counts of molds and yeasts, as well as total coliforms, was reduced in pork burgers [11].

Sodium nitrite/nitrate have been previously widely used to preserve frankfurter-type sausages since, in combination with sodium chloride and thermal treatment, they can inhibit spoilage and the development of pathogen microorganisms [37]. Although chemical preservatives and synthetic antioxidants can be effectively used to improve the shelf life of meat products, the toxicological aspects have become a topic of concern and discussion, because there is no current single substitute for such compounds, particularly for nitrite/nitrate [38]. The use of nitrites as preservatives in meat products is mainly driven by their ability to inhibit the growth of pathogenic bacteria, while helping to maintain the characteristic color and flavor of cured meats. However, there are health concerns surrounding nitrites, especially regarding the formation of nitrosamines, which are potentially carcinogenic and can form when nitrites are exposed to high cooking temperatures [39,40]. This has led to stricter regulations on nitrite levels in processed meats and increased interest in natural nitrite alternatives [41]. In recent years, naturally occurring antimicrobial and antioxidant compounds have been preferably employed in meats because of their potential health benefits and safety compared with synthetic preservatives [42]. The antimicrobial activity of natural extracts is mainly due to phenolic compounds, although their mode of action has not yet been determined. Different mechanisms have been suggested to explain this antimicrobial effect. Hydrophobic phenols are able to penetrate into the phospholipid bilayer and induce several changes in cell functions, including membrane disruption and structural changes [43]. Moreover, they may also be able to enter the cell and deactivate intracellular components such as enzymes [44] or intercalate into microbial DNA [45]; other mechanisms of action include metal chelation [46], protein precipitation [47], or the inactivation of extracellular microbial enzymes [48].

Instrumental color changes in frankfurters at T0 and T1 were significant, according to the formulation (Table 4). In the frankfurters manufactured with RWP, lightness (CIE L*) was significantly reduced by the addition of RWP, while the control and commercial frankfurters presented similar values. At T1, these initial changes remained constant and were not modified during storage. CIE a* (redness) was significantly increased in the commercial group, while the inclusion of RWP decreased the frankfurters’ redness, compared to the control frankfurters. The values of CIE a* increased (*p* < 0.05) during storage in the control frankfurters and those with high levels of RWP. CIE b* (yellowness) was significantly highest in the control formulation, followed by that with low levels of RWP, and then by the commercial formulation; the lowest yellowness values were found in frankfurters with high levels of RWP. During storage, only a slight decrease in yellowness was observed in the control group, and, at T1, the same differences observed at T0 due to the formulation were maintained. Significantly higher chroma values were observed in the control group compared to the other groups, and these values remained unchanged in all groups during storage. On the other hand, the highest hue values were observed in the group with low levels of RWP, while the commercial group had significantly lower values. These differences were maintained at T1.

In the frankfurters manufactured with WWP, the lightness (CIE L*) of the control frankfurters presented the highest values, while the lowest lightness values were found in the commercial frankfurters; the WWP groups (both high and low) presented intermediate lightness values. Lightness values were not modified during storage, and differences at T0 remained constant at T1. Redness (CIE a*) was highest, to a statistically significant level, in the commercial formulation, while the lowest redness values were found in control frankfurters and those made with low levels of WWP; frankfurters made with high levels of WWP presented intermediate values. Initial differences (T0) were maintained at T1, and only small increases in redness were found in control frankfurters and those made with low levels of WWP. Yellowness (CIE b*) was highest, again to a statistically significant level, in the control group, while the lowest yellowness values were observed in the commercial formulation. Similarly, Higuero et al. [49] reported increased CIE a* values and decreased CIE b* values in dry-cured loin, due to the addition of nitrifying salts. This suggests that nitrifying salts are not only responsible for the enhanced redness but also for the reduced yellowness of dry-cured meat products. As found for RWP frankfurters, for WWP frankfurters, the highest chroma values were observed in the control group. At T1, the same differences due to formulation as those found at T0 were maintained. Hue showed a similar pattern, as the highest values were found in the control group. During storage, decreases in hue values were observed in the control and low WWP groups, while they remained constant in the commercial and high WWP groups.

In line with our results, Ryu et al. [50] observed a significant decrease in color lightness in cooked pork sausages fortified with 0.5 and 1% grape pomace powder. The higher CIE a* values in commercial frankfurters that we observed were as expected and resulted from the nitrite salts used, which provides them with their characteristic pink color. The main reason for this is the effect of nitrite on myoglobin, which, during heating, leads to formation of nitrosyl-hemochrome, a pink-colored compound. However, the lower redness values in RWP and WWP frankfurters show that the addition of grape pomace does not result in a red color and cannot compensate for the absence of nitrites, which is in agreement with the literature [51]. In this case, RWP did not result in a bright red color because it consisted not only of pulp and skin but also included seeds, stems, and stalks. Like us, Özvural and Vural [22] found that the CIE a* values of frankfurters were reduced due to the addition of grape seed flour. According to these authors, this decrease may be a result of the suppression of the natural color of the product due to the addition of grape seed flour and/or the change in color of the emulsion due to the different emulsion properties of each treatment. Likewise, Pereira et al. [28] also reported that the CIE a* values of beef burgers initially decreased with red wine grape pomace powder, compared to a control. However, Martín-Mateos et al. [11] and Carrapiso et al. [51] found no effect on color when white grape pomace was added to the formulation of fresh burgers and dry-cured sausages, respectively. Similarly, in dry-cured sausages manufactured with red grape pomace, the redness values were also similar to the values of sausages without grape pomace, since the levels of pomace added were low enough to avoid color modifications in the meat product [52]. Despite the fact that the redness achieved by nitrites was not attained in frankfurters made with pomace, the incorporation of white and red grape pomace generally helped preserve their color during storage. In contrast, the incorporation of white grape pomace into pork burgers did not prevent discoloration during storage [11].

In the frankfurters made with RWP, lipid oxidation (Table 5) showed the highest level in the control batch, the lowest level in the commercial formulation, and intermediate levels in the frankfurters containing RWP. This suggests a potential tendency of the pomace to reduce lipid oxidation. At T1, only low-RWP frankfurters increased the levels of TBA-RS; however, despite this, the lipid oxidation levels of the control frankfurters were significantly higher than those for frankfurters manufactured with pomace and commercial formulations. Therefore, the TBA-RS values of frankfurters made with pomace at T1 were lower than for the control but higher than for the commercial formulation. In addition, the antioxidant effect of the pomace was proportional to the amount of pomace added. On the other hand, protein oxidation was significantly higher in control and commercial than in WWP frankfurters, at both low and high levels. During storage, the initial levels were not modified, and these differences were maintained at T1. Additionally, phenolic compounds levels were highest, to a significant extent, in the commercial formulation. RWP frankfurters (at low and high levels) presented higher phenolic compound levels than the control formulation. These differences among formulations were also detected at T1.

Regarding the effect of WWP, lipid oxidation also showed the highest levels in the control batch, with the lowest levels found in the commercial and WWP formulations. However, during storage, lipid oxidation increased in WWP formulations; at T1, these values were lower than in the control formulation, but higher than in the commercial formulation. Regarding protein oxidation, a similar pattern to that of RWP was found: frankfurters made with WWP had lower levels of protein oxidation than control and commercial frankfurters. During storage, the protein oxidation levels of the commercial formulation were significantly increased, and, at T1, after storage, the commercial group had the highest levels. These results indicate that WWP was able to control the oxidation of lipids and proteins. Regarding phenolic compounds in frankfurters, their levels were highest in the commercial formulation and similar in the control and WWP formulations.

Red and white pomaces had antioxidant effects against lipid oxidation during the manufacturing process of frankfurters (mincing, mixing, heating…) and during the refrigerated storage process of the packaged sausages, although the effect was not as intense as that achieved by the addition of synthetic additives. In contrast, the inclusion of ascorbic acid and nitrites was only effective against lipid oxidation, since the protein oxidation levels of commercial frankfurters underwent similar changes to those of the control frankfurters. Wine pomace is an excellent source of polyphenols [11,53,54]. The highest values for phenols in commercial formulations, when compared to both RWP and WWP frankfurters, could be explained by the fact that the Folin–Ciocalteu reagent used for the quantification of polyphenols may react with the reducing compounds, as well as the phenolics. Therefore, any other reducing substances present in the sample, such as ascorbate, may cause positive interferences, leading to inaccurate results and an overestimation of phenolic content [55]. In previous research, a possible overestimation of the phenolic compound contents due to the Folin–Ciocalteu method has been reported, due to the possible interference of additives, such as sulfite, in burgers [11]. Despite this, frankfurters made with RWP (at both levels) presented higher phenolic contents than the control frankfurters; however, frankfurters made with WWP presented similar contents to the control frankfurters. This is an unexpected result, and differences in the behavior of RWP, with respect to the WWP, may be associated with variations in the phenolic profiles of both pomaces and their resistance to oxygen, temperature, and other conditions.

The effectiveness of grape polyphenols in delaying lipid oxidation in cooked pork and cooked chicken meat has been reported [30,56]. Other studies investigating grape-based products in frankfurter-type sausages also showed that the deterioration of frankfurters in terms of oxidation could be prevented or attenuated by this antioxidant-rich ingredient [22]. The mechanism of the protective effect of grape pomace on lipid oxidation may be due to the presence of a number of oligomer procyanidins, such as catechin and epicatechin [57]. It appears that most of the polyphenols contained in grape skin and seed pomace are hydrophobic, so they can be very effective components for stabilizing free radicals in pork and pork products [58], and they may be of importance for meat emulsions such as frankfurters, in which a hydrophilic phase (water, meat proteins, salts) is emulsified with a lipophilic phase (fat). In our research, we added a stabilized grape pomace from pressed grapes after wine production, including seeds, skin, and pulp, which would explain the antioxidant effect observed in RWP and WWP frankfurters. Previous studies on dry-cured sausages [51] and fresh burgers [11] demonstrated that white grape pomace, stabilized through blanching and HHP, could be utilized to improve the lipid stability of meat products and could be an environmentally friendly alternative to synthetic antioxidants, like metabisulfite in burgers and nitrites in dry-cured products. The antioxidant activity of phenols is primarily due to their ability to donate hydrogen atoms or electrons, thus neutralizing free radicals and preventing oxidative stress [1]. This activity helps to extend the shelf life of food products and enhance their nutritional quality.

On the other hand, the addition of red (RWP) and white (WWP) grape pomace to frankfurters also significantly reduced protein oxidation, as measured by a lower carbonyl content, compared to the control and commercial groups. According to the literature, generally, the efficacy of wine pomace products at inhibiting protein oxidation is lower than against lipid oxidation [1]. However, like us, Jongberg et al. [59] found that white wine pomace extract inhibited the formation of protein carbonyls in beef burgers, and also showed promising protection against protein oxidation in meat during storage. This fact may be relevant for preserving the quality of frankfurters during storage, as the alteration of protein functionality caused by oxidation likely affects their color and texture characteristics [60].

Nitrites, commonly used in commercial frankfurters, are known for their antioxidant properties. However, they did not reduce protein oxidation in frankfurters, showing levels similar to those in the control group. Likewise, previous studies on dry-cured sausages found that the addition of nitrites also failed to decrease protein oxidation [52]. Sodium nitrite can have both anti- and pro-oxidant effects on protein oxidation in meat products, depending on its concentration [61]. Several studies in the literature [62,63] suggest that nitrite’s impact on proteins is complex, acting as both an antioxidant and a prooxidant in proteins, as nitrite is able to initiate oxidation reactions by capturing electrons from molecules that are easily oxidized [64]. Given the significance of oxidative reactions for both the quality of meat products and human health, minimizing these reactions is a top priority for the meat products industry. Lipid oxidation produces harmful compounds like lipid peroxides and free radicals, which can damage biomolecules (RNA, DNA, proteins, lipids, and carbohydrates) and contribute to diseases such as cardiovascular disease, cancer, and neurological disorders, as well as aging [65]. Similarly, protein oxidation is linked to aging and diseases like Alzheimer’s, Parkinson’s, rheumatoid arthritis, and diabetes [66,67].

Table 6 shows the effect of red (RWP) and white (WWP) wine pomace addition on the texture properties of frankfurters. Regarding the effect of RWP, the highest hardness values were observed for the commercial formulation, while the control, low RWP, and high RWP frankfurters presented similar values at T0. No significant effect of pomace addition on the texture parameters of the sausages was observed compared to the control group. The differences in springiness, cohesiveness, chewiness, and resilience were attributed to higher values in the commercial formulation. At T1, the values of all parameters remained unchanged for both the control and the frankfurters manufactured with red wine pomace, while they increased significantly for the commercial formulation in terms of hardness, gumminess, and chewiness. This suggests that the addition of red wine pomace to frankfurters can stabilize most of the textural properties over time, whereas commercial formulations, despite initial advantages, show higher variability during storage.

Regarding the effect of WWP, the addition of this type of pomace generally did not significantly affect the textural properties at T0. Compared to the control, the values for hardness, springiness, cohesiveness, gumminess, chewiness, and resilience were similar. The commercial formulation had the highest values for all texture parameters. In addition, these differences due to the formulation were maintained at T1.

Contrary to our results, Özvural and Vural [23] reported that incorporating 0.5% grape seed extract into frankfurter formulations affected parameters such as hardness and cohesiveness. According to these authors, the hardness of frankfurters made with grape seed extract increased compared to the control, while cohesiveness decreased. The differences observed in comparison to our study may be due to the method of pomace application in the frankfurters (commercial extract vs. ground fresh pomace).

Dong et al. [68] reported that the addition of nitrite to cooked sausages resulted in a more significant increase in hardness and adhesiveness, along with a decrease in elasticity and cohesiveness, as measured by instrumental TPA. This could explain the higher hardness values found in this study in the commercial formulation. Furthermore, this increase in hardness could be related to higher levels of protein oxidation, since some authors have related the hardness of meat to a higher intensity of protein oxidation reactions, leading to the formation of crosslinking and polymerization in proteins [69]. The effect of texture differences at sensory levels was not evaluated, but these changes may affect the acceptability of the developed products.

## 4. Conclusions

The use of Iberian pig fat in the production of frankfurters is a practical approach to improve the fatty acid profiles of these products while utilizing underused raw materials from Iberian meat production. The inclusion of wine pomace marginally decreased the pH of the frankfurters, but this did not offer any significant protection against microbial growth during the manufacturing process. Despite this, sausages made with red or white grape pomace exhibited low levels of lipid and protein oxidation. Notably, both red and white pomace were equally effective in reducing oxidative reactions during frankfurter production, despite the higher initial phenolic compound content and antioxidant activity observed in white wine pomace.

Commercial frankfurters, made with ascorbic acid and nitrites, showed low levels of lipid oxidation but the highest levels of protein oxidation. The lack of efficacy of nitrite in preventing protein oxidation warrants further investigation, particularly to assess its potential negative implications for human health. However, the typical pink color and firm texture of commercial frankfurters were not achieved in the formulations made with pomace, which could make these products less appealing to consumers. Future studies should assess whether these differences significantly impact consumer acceptability.

## Figures and Tables

**Table 1:** Characterization of the valorized wine/grape pomace.

	Red Wine Pomace	White Wine Pomace	*p*-Value
pH	3.94 ± 0.03	3.96 ± 0.03	*ns*
Aw	0.980 ± 0.003	0.971 ± 0.013	*ns*
**Proximate composition** (g 100 g^−1^)			
Moisture	57.4 ± 0.6	69.2 ± 1.5	*ns*
Protein	4.3 ± 0.6	2.3 ± 0.2	*ns*
Fiber	25.4 ± 1.7	18.0 ± 0.9	*
Fat	3.9 ± 0.2	1.7 ± 0.1	**
**Phenolic compounds** (mg 100 g^−1^)	486.0 ± 24.5	766.7 ± 15.6	**
**Antioxidant activity** (mM Trolox mL^−1^)	45.3 ± 3.5	65.9 ± 1.9	**

Significant differences were analyzed using a Student’s *t* test. *ns*: non-significant (*p* > 0.05); * *p* < 0.05; ** *p* < 0.01.

**Table 2:** Proximate composition (g 100 g^−1^), fatty acids profile (%), and pH of frankfurters made with RWP (red wine pomace) and WWP (white wine pomace).

	Control			Commercial			Low Level			High Level			*p*-Formulation	
***Red wine pomace (WWP)***														
Moisture	46.7	±	1.2	46.8	±	2.0	46.1	±	1.8	47.9	±	1.8	*ns*	
Protein	15.1	±	1.4	13.7	±	0.9	13.7	±	1.5	14.5	±	0.4	*ns*	
Fat	20.1	±	3.7	21.2	±	1.7	19.7	±	1.8	22.4	±	1.4	*ns*	
pH	5.94b	±	0.02	5.98a	±	0.01	5.94b	±	0.01	5.86c	±	0.01	***	
**Fatty acids profile (%)**														
Lauric ac. (C12:0)	0.1	±	0.0	0.1	±	0.0	0.1	±	0.0	0.1	±	0.0	*ns*	
Myristic ac. (C14:0)	1.2	±	0.0	1.2	±	0.0	1.2	±	0.1	1.2	±	0.2	*ns*	
Palmitic ac. (C16:0)	22.2	±	0.1	22.1	±	0.2	22.8	±	0.9	22.8	±	1.4	*ns*	
Palmitoleic ac. (C16:1)	1.9	±	0.0	1.9	±	0.0	2.0	±	0.1	1.8	±	0.1	*ns*	
Margaric ac. (C17:0)	0.3	±	0.0	0.2	±	0.0	0.3	±	0.0	0.3	±	0.1	*ns*	
Margaroleic ac. (C17:1)	0.2	±	0.0	0.2	±	0.0	0.2	±	0.0	0.2	±	0.0	*ns*	
Stearic ac. (C18:0)	11.6	±	0.0	11.3	±	0.1	11.7	±	0.3	11.6	±	0.5	*ns*	
Oleic ac. (C18:1)	50.8ab	±	0.1	51.0a	±	0.2	50.0b	±	0.5	50.1ab	±	0.9	*	
Linoleic ac. (C18:2)	9.9	±	0.1	10.0	±	0.1	10.0	±	0.2	10.0	±	0.2	*ns*	
Linolenic ac. (C18:3)	0.4b	±	0.0	0.5ab	±	0.0	0.5a	±	0.0	0.5b	±	0.0	**	
Arachidic ac. (C20:0)	0.1	±	0.0	0.1	±	0.0	0.1	±	0.0	0.1	±	0.0	*ns*	
Gadoleic ac. (C20:1)	1.4	±	0.0	1.4	±	0.0	1.2	±	0.2	1.3	±	0.3	*ns*	
***White wine pomace (WWP)***														
Moisture	44.8	±	1.1	43.0	±	0.6	43.1	±	1.4	43.9	±	0.8	*ns*	
Protein	13.6	±	0.7	13.4	±	0.9	13.3	±	0.8	13.6	±	0.7	*ns*	
Fat	22.2a	±	1.7	19.1b	±	2.1	20.1ab	±	1.1	17.1b	±	1.9	**	
pH	5.90b	±	0.02	5.93a	±	0.01	5.94a	±	0.00	5.86c	±	0.00	***	
**Fatty acids profile (%)**														
Lauric ac. (C12:0)	0.1a	±	0.0	0.1b	±	0.0	0.1b	±	0.0	0.1b	±	0.0	***	
Myristic ac. (C14:0)	1.3a	±	0.0	1.2c	±	0.0	1.2bc	±	0.0	1.2b	±	0.0	***	
Palmitic ac. (C16:0)	27.9a	±	3.3	22.2b	±	0.4	22.5b	±	0.5	22.8b	±	0.2	***	
Palmitoleic ac. (C16:1)	1.9b	±	0.2	1.9ab	±	0.0	2.0b	±	0.1	2.1a	±	0.0	*	
Margaric ac. (C17:0)	0.3a	±	0.0	0.3b	±	0.0	0.3b	±	0.0	0.3b	±	0.0	***	
Margaroleic ac. (C17:1)	0.2	±	0.0	0.2	±	0.0	0.2	±	0.0	0.2	±	0.0	*ns*	
Stearic ac. (C18:0)	14.1a	±	1.8	11.7b	±	0.2	11.5b	±	0.1	11.1b	±	0.1	***	
Oleic ac. (C18:1)	43.3b	±	3.9	50.7a	±	0.5	50.9a	±	0.4	50.3a	±	0.3	***	
Linoleic ac. (C18:2)	9.5	±	0.9	9.8	±	0.1	9.5	±	0.0	10.2	±	0.0	*ns*	
Linolenic ac. (C18:3)	0.5ab	±	0.1	0.5ab	±	0.0	0.4b	±	0.0	0.5a	±	0.0	*	
Arachidic ac. (C20:0)	0.1	±	0.0	0.1	±	0.0	0.1	±	0.0	0.1	±	0.0	*ns*	
Gadoleic ac. (C20:1)	0.9c	±	0.1	1.3a	±	0.0	1.3a	±	0.1	1.2b	±	0.0	***	

*ns*: non-significant (*p* > 0.05); * *p* < 0.05; ** *p* < 0.01; *** *p* < 0.001. Different letters in the same row indicate significant differences in the Tukey test.

**Table 3:** Microbiological counts (log colony forming units; CFU g^−1^) of frankfurters made with RWP (red wine pomace) and WWP (white wine pomace).

	Control			Commercial			Low Level			High Level			*p*-Formulation
***Red wine pomace (RWP)***													
Mesophiles	1.7	±	0.9	2.0	±	0.9	2.4	±	0.6	1.8	±	0.6	*ns*
Psychrophiles	0.9	±	0.5	1.0	±	0.2	1.0	±	0.2	>1			*ns*
Molds and yeasts	1.1	±	0.2	1.1	±	0.3	1.2	±	0.4	0.9	±	0.0	*ns*
*S. aureus*	2.0	±	0.2	<2			2.1	±	0.3	2.1	±	0.2	*ns*
*Cl. perfringens*	<2			<2			<2			<2			*ns*
Total coliforms	1.1	±	0.4	<1			0.9	±	0.0	<1			*ns*
*E. coli*	<1			<1			0.9	±	0.0	<1			*ns*
***White wine pomace (WWP)***													
Mesophiles	2.5	±	0.5	2.2	±	0.2	2.9	±	0.5	2.9	±	0.7	*ns*
Psychrophiles	<1			<1			1.1	±	0.2	1.0	±	0.3	*ns*
Molds and yeasts	*1.2*	±	0.6	<1			1.0	±	0.0	1.1	±	0.4	*ns*
*S. aureus*	2.1	±	0.2	2.0	±	0.2	2.1	±	0.2	<2			*ns*
*Cl. perfringens*	<2			<2			<2			<2			*ns*
Total coliforms	<1			<1			0.9	±	0.0	1.2	±	0.6	*ns*
*E. coli*	<1			<1			<1			<1			*ns*

*ns*: non-significant (*p* > 0.05).

**Table 4:** Instrumental color changes in frankfurter sausages made with RWP (red wine pomace) and WWP (white wine pomace).

	Storage (Days)	Control			Commercial			Low Level			High Level			*p*-Formulation
***Red wine pomace (RWP)***														
L*	T0	69.9a	±	1.5	69.6a	±	0.9	65.7b	±	1.9	59.9c	±	0.6	***
	T1	68.4a	±	1.3	70.4a	±	1.0	68.5a	±	2.0	60.3b	±	0.5	***
	*p-storage*	*ns*			*ns*			*ns*			*ns*			
a*	T0	2.0b	±	0.1	8.3a	±	0.2	1.4c	±	0.1	1.9b	±	0.1	***
	T1	2.4b	±	0.1	8.3a	±	0.2	1.7d	±	0.3	2.1c	±	0.1	***
	*p-storage*	***			*ns*			*ns*			*			
b*	T0	14.3a	±	0.1	10.8c	±	0.2	12.3b	±	0.6	8.7d	±	0.2	***
	T1	14.0a	±	0.3	10.5c	±	0.2	11.6b	±	0.3	8.5d	±	0.2	***
	*p-storage*	*			*ns*			*ns*			*ns*			
Chroma	T0	14.4a	±	0.1	13.6b	±	0.3	12.3c	±	0.6	8.9d	±	0.2	***
	T1	14.2a	±	0.3	13.4b	±	0.2	11.8c	±	0.3	8.8d	±	0.2	***
	*p-storage*	*ns*			*ns*			*ns*			*ns*			
Hue	T0	82.1b	±	0.4	52.5d	±	0.3	83.4a	±	0.3	77.5c	±	0.7	***
	T1	80.1b	±	0.2	51.9d	±	0.4	81.6a	±	1.3	76.3c	±	0.7	***
	*p-storage*	***			*			*			*			
***White wine pomace (WWP)***														
L*	T0	71.5a	±	1.1	66.7c	±	0.9	68.6b	±	1.7	66.3c	±	0.7	***
	T1	72.5a	±	0.9	67.4c	±	1.2	69.5b	±	0.7	66.5c	±	0.8	***
	*p-storage*	*ns*			*ns*			*ns*			*ns*			
a*	T0	1.5c	±	0.1	8.4a	±	0.4	1.7c	±	0.0	2.7b	±	0.1	***
	T1	1.7d	±	0.2	8.2a	±	0.5	2.2c	±	0.2	2.8b	±	0.1	***
	*p-storage*	**			*ns*			***			*ns*			
b*	T0	14.3a	±	0.1	9.9d	±	0.2	12.9c	±	0.3	13.7b	±	0.1	***
	T1	14.0a	±	0.3	10.1d	±	0.2	12.7c	±	0.1	13.5b	±	0.3	***
	*p-storage*	*ns*			*ns*			*ns*			*ns*			
Chroma	T0	14.4a	±	0.1	13.0c	±	0.4	13.0c	±	0.3	13.9b	±	0.1	***
	T1	14.1a	±	0.3	13.1b	±	0.3	12.9b	±	0.1	13.8a	±	0.3	***
	*p-storage*	*ns*			*ns*			*ns*			*ns*			
Hue	T0	84.2a	±	0.3	49.7d	±	0.9	82.6b	±	0.1	78.9c	±	0.2	***
	T1	83.0a	±	0.7	51.0d	±	2.0	80.3b	±	0.9	78.1c	±	0.8	***
	*p-storage*	**			*ns*			***			*ns*			

ns: non-significant (*p* > 0.05); * *p* < 0.05; ** *p* < 0.01; *** *p* < 0.001. T0: 1 day after manufacturing; T1: after 45 days of storage at 5 °C. Different letters in the same row indicate significant differences in the Tukey test.

**Table 5:** Lipid (mg MDA kg^−1^) and protein oxidation (nmols carbonyls mg protein^−1^) changes and phenolic compound contents (mg 100 g^−1^) changes in frankfurters made with RWP (red wine pomace) and WWP (white wine pomace).

	Storage (Days)	Control			Commercial			Low Level			High Level			*p*-Formulation
***Red wine pomace (RWP)***														
Lipid oxidation	T0	1.0a	±	0.1	0.1c	±	0.0	0.2b	±	0.0	0.1ab	±	0.0	***
	T1	1.0a	±	0.1	0.0d	±	0.0	0.4b	±	0.0	0.1c	±	0.0	***
	*p-storage*	*ns*			*ns*			***			*ns*			
Protein oxidation	T0	2.7a	±	0.6	2.5a	±	0.5	1.7b	±	0.2	1.6b	±	0.3	***
	T1	2.7a	±	0.6	2.7a	±	0.4	1.8b	±	0.4	1.7b	±	0.1	***
	*p-storage*	*ns*			*ns*			*ns*			*ns*			
Phenolic compounds	T0	10.4c	±	1.3	60.3a	±	3.6	15.1b	±	1.2	18.1b	±	0.3	***
	T1	11.6d	±	1.3	69.3a	±	3.2	15.0c	±	1.4	22.5b	±	1.1	***
	*p-storage*	*ns*			**			*ns*			***			
***White wine pomace(WWP)***														
Lipid oxidation	T0	0.6a	±	0.1	0.0b	±	0.0	0.2b	±	0.0	0.1b	±	0.0	***
	T1	0.6a	±	0.0	0.1d	±	0.1	0.4b	±	0.0	0.2c	±	0.0	***
	*p-storage*	*ns*			*ns*			***			***			
Protein oxidation	T0	2.5a	±	0.5	2.3a	±	0.2	1.7b	±	0.4	1.5b	±	0.2	***
	T1	1.8b	±	0.3	5.6a	±	0.6	2.5b	±	1.0	2.2b	±	0.5	***
		*			***			ns			*			
Phenolic compounds	T0	17.6b	±	3.6	67.6a	±	5.0	14.4b	±	1.6	19.4b	±	3.1	***
	T1	14.b	±	3.2	42.6a	±	3.9	13.7b	±	2.1	13.2b	±	2.3	***
	*p-storage*	***			***			*ns*			**			

*ns*: non-significant (*p* > 0.05); * *p* < 0.05; ** *p* < 0.01; *** *p* < 0.001. T0: 1 day after manufacturing; T1: after 45 days of storage at 5 °C. Different letters in the same row indicate significant differences in the Tukey test.

**Table 6:** Texture parameters changes in the frankfurters manufactured with RWP (red wine pomace) and WWP (white wine pomace).

	Storage (Days)	Control			Commercial			Low Level			High Level			*p*-Formulation
	***Red wine pomace (RWP)***													
Hardness (N cm^2^)	T0	15.8ab	±	1.5	17.1ab	±	0.2	14.6b	±	1.9	17.8a	±	0.5	*
	T1	19.6b	±	2.6	25.5a	±	2.8	16.7b	±	0.8	19.0	±	2.2	**
	*p-storage*	*ns*			**			*ns*			*ns*			
Springiness (cm)	T0	0.8ab	±	0.0	0.9a	±	0.0	0.8ab	±	0.0	0.7b	±	0.0	*
	T1	0.9	±	0.0	0.9	±	0.0	0.9	±	0.0	0.9	±	0.0	*ns*
	*p-storage*	*ns*			*ns*			**			*			
Cohesiveness	T0	0.4b	±	0.0	0.5a	±	0.0	0.4ab	±	0.0	0.4b	±	0.0	*
	T1	0.4b	±	0.0	0.5a	±	0.0	0.4bc	±	0.0	0.3c	±	0.0	**
	*p-storage*	*ns*			*ns*			*ns*			*ns*			
Gumminess (N cm s^2^)	T0	6.0	±	1.0	7.8	±	0.6	5.8	±	1.1	6.4	±	0.1	*ns*
	T1	8.0b	±	0.8	12.3a	±	1.8	6.4b	±	0.4	6.5b	±	0.8	**
	*p-storage*	*ns*			*			*ns*			*ns*			
Chewiness (N cm s^2^)	T0	4.8b	±	0.7	6.6a	±	0.6	4.6b	±	0.8	4.7b	±	0.3	*
	T1	6.8b	±	0.5	10.9a	±	1.9	5.6b	±	0.5	5.6b	±	0.8	**
	*p-storage*	*			*			*ns*			*ns*			
Resilience	T0	0.1b	±	0.0	0.2a	±	0.0	0.1b	±	0.0	0.1b	±	0.0	**
	T1	0.2b	±	0.0	0.2a	±	0.0	0.2b	±	0.0	0.1b	±	0.0	**
	*p-storage*	*ns*			*ns*			*ns*			*ns*			
	***White wine pomace (WWP)***													
Hardness (N cm^2^)	T0	17.8b	±	0.5	20.4a	±	1.0	17.7b	±	1.0	17.6b	±	0.1	*ns*
	T1	23.5b	±	0.5	28.7a	±	0.7	23.7b	±	0.5	23.2b	±	0.1	***
	*p-storage*	***			***			**			***			
Springiness (cm)	T0	0.8	±	0.0	0.9	±	0.0	0.8	±	0.0	0.9	±	0.0	*ns*
	T1	0.9	±	0.0	0.9	±	0.1	0.9	±	0.0	0.9	±	0.0	*ns*
	*p-storage*	*ns*			*ns*			*ns*			*ns*			
Cohesiveness	T0	0.5b	±	0.0	0.6a	±	0.0	0.4b	±	0.0	0.4b	±	0.0	**
	T1	0.5bc	±	0.0	0.6a	±	0.0	0.5b	±	0.0	0.4c	±	0.0	***
	*p-storage*	*ns*			*ns*			*			*ns*			
Gumminess (N cm s^2^)	T0	8.6b	±	1.0	11.7a	±	0.7	7.0b	±	0.3	7.5b	±	0.5	***
	T1	11.1b	±	0.6	16.8a	±	0.4	12.1b	±	0.4	10.1c	±	0.3	***
	*p-storage*	*			***			***			**			
Chewiness (N cm s^2^)	T0	7.2b	±	0.7	10.7a	±	1.37b	5.9b	±	0.2	6.2b	±	0.3	***
	T1	9.5b	±	0.9	15.2a	±	0.6	10.3b	±	0.7	8.9b	±	0.5	***
	*p-storage*	*			**			**			**			
Resilience	T0	0.2b	±	0.0	0.3a	±	0.0	0.2b	±	0.0	0.2b	±	0.0	***
	T1	0.2c	±	0.0	0.3a	±	0.0	0.2b	±	0.0	0.2c	±	0.0	***
	*p-storage*	*ns*			*ns*			**			*ns*			

*ns*: non-significant (*p* > 0.05); * *p* < 0.05; ** *p* < 0.01; *** *p* < 0.001. T0: 1 day after manufacturing; T1: after 45 days of storage at 5 °C. Different letters in the same row indicate significant differences in the Tukey test.

## Data Availability

The original contributions presented in the study are included in the article; any further inquiries can be directed to the corresponding author.

## Supplementary Materials

The following supporting information can be downloaded at: https://www.mdpi.com/article/10.3390/foods14030391/s1: Table S1: Fatty acid profile (%) of Iberian pork fat used for the preparation of the frankfurters.
