# Supplementary material for: Frankfurters Manufactured with Valorized Grape Pomace as a Substitute of Nitrifying Salts
Source: Foods. 2025 Jan 24;14(3):391. doi: 10.3390/foods14030391 (PMC11816958; doi:10.3390/foods14030391)
Supplement: Supplementary file 1 [file foods-14-00391-s001.zip › foods-3406627-supplementary.pdf]

Table 1 S. Fatty acid profile (%) of Iberian pork fat used for the preparation of the frankfurters.

	Mean±SD		
Lauric ac. (C12:0)	0.1	±	0.0
Myristic ac. (C14:0)	1.3	±	0.0
Palmitic ac. (C16:0)	22.0	±	0.2
Palmitoleic ac. (C16:1)	1.8	±	0.0
Margaric ac. (C17:0)	0.3	±	0.0
Margaroleic ac. (C17:1)	0.3	±	0.0
Stearic ac. (C18:0)	11.7	±	0.0
Oleic ac. (C18:1)	51.0	±	0.1
Linoleic ac. (C18:2)	9.3	±	0.0
Linolenic ac. (C18:3)	0.6	±	0.0
Arachidic ac. (C20:0)	0.2	±	0.0
Gadoleic ac. (C20:1)	1.5	±	0.1
